# LC–MS/MS Quantification of the Anti-Tuberculosis Macrolide Arenicolide A in Rat Plasma: Method Validation and Pharmacokinetic Application

**DOI:** 10.3390/pharmaceutics18101205

**Published:** 2026-09-22

**Authors:** Dahan Kim, Eun Kyung Shin, Seong-Wook Kang, Min-Jae Kim, Na-Young Yu, Pil-Jae Song, Min-Jun Baek, Sunghoon Hwang, Thanh-Hau Huynh, Soohyun Um, Yun Kwon, Dong-Chan Oh, Dae-Duk Kim

**Affiliations:** 1 College of Pharmacy, Seoul National University, Gwanak-gu, Seoul 08826, Republic of Korea; dahankim@snu.ac.kr (D.K.); eun8sun@snu.ac.kr (E.K.S.); tjddnr3570@snu.ac.kr (S.-W.K.); alswo7725@snu.ac.kr (M.-J.K.); nyyu8520@snu.ac.kr (N.-Y.Y.); vlfwo117@snu.ac.kr (P.-J.S.); sh1646@princeton.edu (S.H.); thanhhaud2012@gmail.com (T.-H.H.); dongchanoh@snu.ac.kr (D.-C.O.); 2 Research Institute of Pharmaceutical Sciences, Seoul National University, Gwanak-gu, Seoul 08826, Republic of Korea; 3 Department of Bio and Brain Engineering, Korea Advanced Institute of Science and Technology (KAIST), Daejeon 34141, Republic of Korea; baek.mj@kaist.ac.kr; 4 Natural Products Research Institute, Seoul National University, Gwanak-gu, Seoul 08826, Republic of Korea; 5 Department of Forest Products and Biotechnology, Kookmin University, Seoul 02707, Republic of Korea; drums@kookmin.ac.kr; 6 College of Pharmacy and Research Institute of Pharmaceutical Sciences, Kyungpook National University, Daegu 41566, Republic of Korea; yunkwon@knu.ac.kr

**Keywords:** arenicolide A, LC–MS/MS, bioanalytical method validation, pharmacokinetics

## Abstract

**Background/Objectives**: Arenicolide A (ArA), a 26-membered macrolide from *Micromonospora* sp. GR10, is active against *Mycobacterium tuberculosis*, including multidrug-resistant strains. We developed and validated a liquid chromatography–tandem mass spectrometry (LC–MS/MS) method for ArA in rat plasma and applied it to pharmacokinetic studies. **Methods**: Plasma (50 µL) was processed by protein precipitation, and analyzed using 4 min isocratic C_18_ chromatography with positive-ion multiple reaction monitoring (*m*/*z* 827.5 → 765.5 for ArA; *m*/*z* 237.1 → 194.1 for carbamazepine, internal standard). Validation was performed with reference to the International Council for Harmonisation M10 guideline. Pharmacokinetics were evaluated in male Sprague–Dawley rats after intravenous (1 mg/kg) or oral (5 or 10 mg/kg) administration. Passive permeability, aqueous solubility and rat microsomal depletion were also evaluated. **Results**: The assay was linear over 2.0–2000 ng/mL (*r*^2^ > 0.996), with a lower limit of quantification of 2.0 ng/mL. Within- and between-run relative errors were −5.81 to 2.30% and −3.20 to −0.10%, with precision ≤9.01% and ≤10.38%. Extraction recovery was 87.7–96.5%, and matrix effects were consistent across six plasma lots. ArA was stable under pre- and post-preparative conditions. Following intravenous administration, ArA showed a terminal half-life of 0.78 ± 0.08 h and plasma clearance of 26.1 ± 2.5 mL/min/kg. Absolute oral bioavailability was 16.8% and 14.2% at oral doses of 5 and 10 mg/kg, respectively. ArA exhibited high passive permeability, limited aqueous solubility of approximately 0.1 mg/mL, and slow microsomal depletion. **Conclusions**: This validated method enabled characterization of ArA pharmacokinetics and oral bioavailability. This method supports a reliable analytical platform for further formulation and preclinical evaluation of ArA as an anti-tuberculosis lead.

## 1. Introduction

Tuberculosis (TB) remains a leading cause of mortality from infectious diseases, and the continued emergence and transmission of multidrug-resistant TB (MDR-TB) undermine the effectiveness of established treatment regimens [1,2,3,4]. Natural products remain an important source of structurally diverse scaffolds for anti-infective drug discovery [5,6,7]. Arenicolide A (ArA) is a 26-membered macrolide originally isolated from the marine actinomycete *Salinispora arenicola* and subsequently identified among the metabolites of *Micromonospora* sp. GR10, which was obtained from the gut of the female black oil beetle *Meloe proscarabaeus* [8,9]. ArA inhibits *Mycobacterium tuberculosis* (Mtb), with a half-minimum inhibitory concentration (MIC_50_) of 0.8 μM; retains activity against multidrug-resistant and extensively drug-resistant strains and intracellular Mtb; and has demonstrated efficacy in a murine model of MDR-TB infection [9].

Initial intravenous and oral pharmacokinetic (PK) studies in C3HeB/FeJ mice demonstrated rapid absorption and measurable systemic exposure following oral administration [9]. However, validation data demonstrating the performance of the bioanalytical assay used in those studies were not reported, limiting assessment of their pharmacokinetic parameters. Because reliable characterization of plasma PK and absolute oral bioavailability requires an assay validated for selectivity, sensitivity, calibration performance, accuracy, precision, matrix effect, and stability in the relevant biological matrix [10], the absence of such a validated assay for ArA constitutes the principal gap addressed in the present work.

The development of such an assay for ArA presents several analytical challenges. Because its structure lacks a strongly basic protonation site, the protonated molecules were not expected to provide adequate sensitivity under positive electrospray ionization, necessitating the evaluation of alternative precursor ions [8,11,12]. In addition, a stable-isotope-labeled form of ArA was not commercially available, requiring the selection and evaluation of an alternative internal standard (IS). These considerations necessitated the optimization of sample preparation, chromatographic separation, and mass spectrometric detection.

In the present study, an LC–MS/MS method for quantifying ArA in rat plasma was developed and validated with reference to the International Council for Harmonisation (ICH) M10 guideline [10]. Then, the method was applied to characterize the plasma PK of ArA and to estimate its absolute oral bioavailability in male Sprague–Dawley rats. As supporting assessments, passive membrane permeability, apparent aqueous solubility, and apparent depletion in NADPH-supplemented rat liver microsomes were evaluated to provide preliminary context for the observed oral exposure. This work thereby establishes a validated analytical basis for the PK evaluation of ArA and supports its continued preclinical development as a potent anti-tuberculosis agent.

## 2. Materials and Methods

### 2.1. Materials

ArA was isolated from the culture extract of *Micromonospora* sp. GR10, purified by reversed-phase C_18_ high-performance liquid chromatography (HPLC), and structurally characterized by one- and two-dimensional nuclear magnetic resonance spectroscopy and high-resolution fast atom bombardment mass spectrometry, as described previously [9]. Carbamazepine (CBZ, purity ≥ 98%), nadolol (purity ≥ 98%), 1,2-dioleoyl-sn-glycero-3-phosphocholine (DOPC) and n-dodecane were purchased from Sigma-Aldrich (St. Louis, MO, USA). Metoprolol tartrate (purity ≥ 98%) and ketoprofen (purity ≥ 98%) were obtained from Tokyo Chemical Industry (Tokyo, Japan). β-Nicotinamide adenine dinucleotide phosphate, reduced form (NADPH)-regenerating system consisting of NADP^+^, glucose-6-phosphate, glucose-6-phosphate dehydrogenase, and magnesium chloride was purchased from Thermo Fisher Scientific (Waltham, MA, USA). Pooled male Sprague–Dawley rat liver microsomes (20 mg/mL) were purchased from XenoTech, LLC (Kansas City, KS, USA) and stored at −80 °C until use. HPLC-grade methanol, water, and acetonitrile (ACN) were purchased from Thermo Fisher Scientific (Waltham, MA, USA). MultiScreen^®^ 96-well filter plates equipped with hydrophobic polyvinylidene fluoride (PVDF) membranes (pore size, 0.45 μm; effective filtration area, 0.3 cm^2^; catalog no. MAIPN4550) and 96-well collection plate were obtained from Merck Millipore (Burlington, MA, USA). The filter membrane served as a support for the artificial lipid membrane. All other reagents were of analytical grade.

### 2.2. Animal Studies

Rats were selected to permit serial blood sampling through an indwelling arterial cannula, thereby allowing a complete concentration–time profile to be obtained within each animal. Because each animal provided a complete individual profile, three animals were included per group to obtain preliminary individual profiles while limiting animal use. Male Sprague–Dawley rats (8 weeks old, 250 ± 10 g) were obtained from Koatech Inc. (Pyeongtaek, Republic of Korea) and acclimatized for 7 days before use. Animals were housed three per cage under controlled temperature (22 ± 2 °C) and humidity (55 ± 5%) conditions with a 12 h light/dark cycle, with free access to standard rodent chow and water. A total of nine rats were used and were assigned at random to the intravenous and oral dose groups. At the end of the sampling period, animals were euthanized by CO_2_ inhalation, followed by cervical dislocation. All animal procedures were reviewed and approved by the Institutional Animal Care and Use Committee of Seoul National University (approval no. SNU-221114-2-7) and were performed in accordance with institutional guidelines for the care and use of laboratory animals.

### 2.3. Preparation of Calibration Standard and Quality Control Samples

Stock solutions of ArA (1.0 mg/mL in methanol) and CBZ (1.0 mg/mL in ACN) were prepared by vortex mixing. CBZ was used as IS. The stock solution of ArA was serially diluted with methanol to prepare working solutions at concentrations 20 times higher than the intended concentrations for the calibration standards and quality control (QC) samples (concentration range: 0.04–40 µg/mL). A working solution of CBZ was also prepared in ACN at a concentration of 200 ng/mL. Calibration standards were prepared by adding 5 µL of each ArA working solution to 95 µL of blank rat plasma, yielding 100 µL of spiked plasma with a final organic solvent content of 5% (*v*/*v*) and final concentrations of 2.0, 5.0, 10, 30, 100, 200, 400, 1000, and 2000 ng/mL. The QC samples were prepared separately in the same manner at the concentrations of 5.0, 925, and 1850 ng/mL. A 50 µL aliquot of each calibration standard and QC sample was then processed as described in Section 2.4. The concentration of the QC samples was selected based on the ICH M10 guideline: the low QC within 3 times the lower limit of quantification (LLOQ), the medium QC at approximately 30% to 50% of the calibration range, and the high QC at no less than 75% of the upper limit of quantification (ULOQ).

### 2.4. Sample Pretreatment

The protein precipitation method was used for pretreatment of analytical samples. To a 50 µL aliquot of plasma samples (i.e., blank plasma, calibration standards, or QC samples), 10 µL of IS working solution and 190 µL of ACN were added and vortex-mixed for 7 min to induce protein precipitation. The samples were then centrifuged at 16,000× *g* for 5 min. The supernatant was collected into a sample vial and placed in the autosampler of the LC–MS/MS system for analysis.

### 2.5. LC–MS/MS Analysis of ArA

The quantification of ArA was conducted by liquid chromatography–tandem mass spectrometry (LC–MS/MS) using an Agilent 1260 Infinity HPLC system coupled to an Agilent 6430 Triple Quadrupole LC/MS system equipped with an electrospray ionization (ESI) source (Agilent Technologies, Santa Clara, CA, USA). The HPLC system comprised a G1312B binary pump, a G1367E autosampler, a G1316C thermostated column compartment, and a G1330B thermostat; the autosampler was maintained at 10 °C throughout the analysis. An aliquot (5 µL) of the pretreated sample was injected onto an Agilent Poroshell 120 EC-C_18_ column (2.7 µm, 4.6 × 50 mm) and column temperature was maintained at 40 °C. Chromatographic separation of ArA was achieved using a mobile phase consisting of 10 mM ammonium formate in water and ACN (20:80, *v*/*v*). Isocratic elution was conducted at a flow rate of 0.4 mL/min over a run time of 4 min.

Mass spectrometric detection was conducted in positive ESI mode under optimized conditions. The optimized ESI source settings were as follows: gas temperature, 350 °C; gas flow, 11 L/min; nebulizer pressure (nitrogen), 60 psi; and capillary voltage, 4.00 kV. The multiple reaction monitoring (MRM) mode was used at unit resolution for both mass filters (Q1 and Q3). The molecular mass transitions of the precursor ion to product ion/fragmentor voltages (V)/collision energies (eV) for ArA and CBZ were *m*/*z* 827.5 → 765.5/105 V/32 eV and *m*/*z* 237.1 → 194.1/126 V/18 eV, respectively. Data acquisition and processing were performed using MassHunter Workstation Software Quantitative Analysis (Version B.05.00; Agilent Technologies).

### 2.6. Method Validation

Validation procedures for the analysis of ArA in rat plasma were conducted with reference to the ICH M10 guideline [10].

#### 2.6.1. Selectivity and Carry-Over

The selectivity of the analytical method was assessed by comparing chromatograms of blank plasma samples collected from six individual rats. Responses in the acquisition windows of ArA and the IS were considered acceptable if they did not exceed 20% of the mean ArA response at the LLOQ and 5% of the mean IS response. Carry-over was investigated by analyzing blank samples immediately after the calibration standard at the ULOQ (2000 ng/mL) and was evaluated against the same criteria.

#### 2.6.2. Linearity and Lower Limit of Quantification

The linearity of the calibration curve was evaluated over a concentration range of 2.0–2000 ng/mL. The curves were constructed by plotting the response ratios of ArA to the IS against the corresponding nominal concentrations of the calibration standards. This analysis employed least-squares regression with a weighting factor of 1/x. Linearity was evaluated based on the coefficients of determination (*r*^2^) of the calibration curve. The LLOQ was defined as the lowest concentration that could be quantified with acceptable accuracy and precision, characterized by a relative error (RE) within ±20% and a relative standard deviation (RSD) not greater than 20%.

#### 2.6.3. Within- and Between-Run Accuracy and Precision

Six replicates of the LLOQ (2.0 ng/mL) and QC samples (low QC: 5.0 ng/mL; medium QC: 925 ng/mL; and high QC: 1850 ng/mL) were analyzed in three different runs on three consecutive days to assess both within- and between-run accuracy and precision. Accuracy was assessed based on RE, with the acceptable range of ±15% for the QC samples and ±20% for the LLOQ samples. Precision was determined using RSD, with the acceptable thresholds of ≤15% for the QC samples and ≤20% for the LLOQ samples.

#### 2.6.4. Matrix Effect and Extraction Recovery

The matrix effect was evaluated in accordance with the ICH M10 guideline, by analyzing three replicates of low and high QCs, each prepared using matrix obtained from six different rats. For each individual matrix source, accuracy and precision were assessed using RE and RSD, with acceptance criteria of ±15% and ≤15%, respectively. The extraction recoveries of the analytes were evaluated by comparing the detection response of analytes spiked before extraction with those spiked after the extraction process for three replicates.

#### 2.6.5. Pre- and Post-Preparative Stability

The stability of ArA was evaluated under four distinct conditions that the analytes may encounter during the analytical process. To assess pre-preparative stability, four replicates of low, medium and high QC samples were prepared. One set was stored at room temperature for 90 min to simulate sample preparation conditions, while another set was maintained at −20 °C for 35 days to evaluate long-term stability. The third set underwent three freeze–thaw cycles between −20 °C and 4 °C to examine freeze–thaw stability. Additionally, post-preparative stability was assessed by keeping the analytical samples in the autosampler at 10 °C for 24 h.

#### 2.6.6. Dilution Integrity

To evaluate dilution integrity, plasma samples with ArA concentrations of 5000 ng/mL were prepared by the method described in Section 2.3. The samples were diluted 5- and 10-fold with the blank rat plasma and subjected to the protein precipitation method described in Section 2.4. The accuracy and precision of the calculated concentration were assessed in terms of RE and RSD for six replicates, with an acceptable range of ±15% and ≤15%, respectively.

### 2.7. In Vivo Pharmacokinetic Study

An in vivo pharmacokinetic study of ArA was conducted in male Sprague–Dawley rats. ArA was dissolved sequentially in ethanol and polyethylene glycol (PEG) 400 and diluted with normal saline to a final composition of 5:80:15 (*v*/*v*/*v*). The rats were fasted overnight before administration, with free access to water. Under anesthesia with Zoletil (40 mg/kg, intramuscular; Virbac Korea Co., Ltd., Seoul, Republic of Korea), the left femoral artery and vein were cannulated using polyethylene tubes (Intramedic™ PE-50; Becton Dickinson Diagnostics, Franklin Lakes, NJ, USA). ArA was administered intravenously through the femoral vein at 1 mg/kg in a dose volume of 2 mL/kg (0.5 mg/mL solution, *n* = 3) or by oral gavage at 5 or 10 mg/kg in a dose volume of 4 mL/kg (1.25 mg/mL or 2.5 mg/mL solution, respectively, *n* = 3 per group). Blood samples (250 μL) were collected through the femoral artery at 5, 15, 30, 60, 120, and 240 min after dosing; for the intravenous groups, an additional sample was collected at 1 min to characterize the initial distribution phase. After each sampling, an equal volume of heparinized normal saline was infused. The blood samples were centrifuged at 16,000× *g* for 5 min at 4 °C. The resulting plasma samples were stored at −20 °C until analysis and were analyzed within 35 days of collection, a period covered by the long-term stability evaluation described in Section 2.6.5. Before LC–MS/MS analysis, 50 μL aliquots of the resulting plasma were processed and deproteinized as described in Section 2.4. Plasma concentrations of ArA were determined by the analytical method described in Section 2.5. Samples with concentrations exceeding the upper limit of quantification were diluted with blank rat plasma using the dilution factors validated in Section 2.6.6 and reanalyzed.

The pharmacokinetic parameters of ArA were calculated by noncompartmental analysis in WinNonlin software (version 5.0.1, Certara, Princeton, NJ, USA). The terminal rate constant was estimated by log-linear regression of the last three observed concentrations, and the terminal half-life (t_1/2_) was calculated as ln(2) divided by this constant. The area under the plasma concentration–time curve from 0 to the last sampling time (AUC_last_), the area under the curve extrapolated to infinity (AUC_inf_), systemic plasma clearance (CL_p_), volume of distribution at steady state (V_d,ss_), the maximum plasma concentration (C_max_), and the time to reach C_max_ (T_max_) were obtained from the plasma concentration data. Absolute oral bioavailability (F, expressed as a percentage) was calculated as the ratio of the dose-normalized group mean AUC_inf_ after oral administration to the dose-normalized group mean AUC_inf_ after intravenous administration.

### 2.8. In Vitro Physicochemical and Metabolic Characterization

To provide preliminary context for the oral exposure of ArA observed in vivo pharmacokinetic studies, passive membrane permeability, apparent aqueous solubility, and apparent parent depletion in rat liver microsomes were determined in vitro.

#### 2.8.1. Parallel Artificial Membrane Permeability Assay

The passive membrane permeability of ArA was assessed by parallel artificial membrane permeability assay (PAMPA) using nadolol, metoprolol, and ketoprofen as low-permeability, low/high permeability boundary, and high-permeability reference compounds, respectively [13,14,15,16]. DOPC was dissolved in n-dodecane at 20 mg/mL, and 5 µL of the lipid solution was applied to each hydrophobic PVDF membrane of the MultiScreen^®^ filter plate. Stock solutions of nadolol, metoprolol, ketoprofen, and ArA were prepared in dimethyl sulfoxide (DMSO) at concentrations of 2, 4, 4, and 0.4 mM, respectively. Each stock solution was diluted 20-fold with phosphate-buffered saline (PBS), previously adjusted to pH 6.5 with hydrochloric acid, to obtain donor solutions containing nadolol, metoprolol, ketoprofen, and ArA at final concentrations of 100, 200, 200, and 20 μM, respectively. The final DMSO concentration in all donor solutions was 5% (*v*/*v*). The donor wells were filled with 300 μL of the respective analyte solution, with eight replicate wells per compound on the same plate. The acceptor wells were filled with 150 μL of PBS (pH 7.4) containing 5% (*v*/*v*) DMSO. The donor and acceptor plates were assembled, covered, and incubated at 25 °C for 10 h in an orbital shaker with continuous agitation at 50 rpm. Following incubation, samples were collected from the acceptor compartments for quantitative analysis.

Ketoprofen concentrations in the acceptor samples were determined by measuring absorbance at 260 nm using a Multiskan microplate spectrophotometer (Thermo Fisher Scientific, Waltham, MA, USA). Nadolol, metoprolol, and ArA samples were processed according to the sample preparation procedure described in Section 2.4 and subsequently analyzed by LC–MS/MS. Calibration standards were prepared in blank acceptor medium consisting of PBS (pH 7.4) containing 5% (*v*/*v*) DMSO and were processed in the same manner as the corresponding PAMPA samples. For nadolol and metoprolol, the chromatographic and electrospray ionization source conditions were identical to those used for ArA analysis. Nadolol and metoprolol were monitored using precursor-to-product ion transitions of *m*/*z* 310.2 → 254.2 and *m*/*z* 268.2 → 191.1, respectively. The fragmentor voltages were 102 V for nadolol and 91 V for metoprolol, and the corresponding collision energies were 17 and 15 eV, respectively.

The apparent permeability coefficient (*P*_app_, cm/s) was calculated from the acceptor concentration using the following equation:

$$P_{\mathrm{app}}=-\frac{V_{D}V_{A}}{\left(V_{D}+V_{A}\right)At}\ln\left[1-\frac{V_{D}+V_{A}}{V_{D}}\frac{C_{A}\left(t\right)}{C_{0}}\right]$$

*V*_D_ and *V*_A_ are the donor and acceptor volumes, 0.30 and 0.15 cm^3^, respectively, *A* is the effective membrane area 0.3 cm^2^, *t* is the incubation time 36,000 s, *C*_A_(*t*) is the analyte concentration in the acceptor compartment after incubation, and *C*_0_ is the initial analyte concentration in the donor compartment.

#### 2.8.2. Aqueous Solubility Study

The apparent equilibrium solubility of ArA was determined in PBS (pH 7.4), double-distilled water (DDW), and normal saline using the saturation shake-flask method. An excess amount of ArA (approximately 0.5 mg) was added to 0.5 mL of each medium in a sealed microcentrifuge tube, with three independent tubes prepared per medium. The samples were vortex-mixed for 1 min and agitated at 50 rpm for 24 h at 37 °C in a shaking water bath. After equilibration, the samples were immediately centrifuged at 16,000× *g* for 15 min, and the clear supernatants were carefully collected without disturbing the solid pellet. Aliquots of the supernatants were diluted as necessary and processed with the IS working solution and ACN as described in Section 2.4. ArA concentrations were determined using the LC–MS/MS method described in Section 2.5 with matrix-matched calibration standards prepared in the corresponding aqueous medium.

#### 2.8.3. ArA Depletion in Rat Liver Microsomes

The apparent depletion of ArA was evaluated using pooled male Sprague–Dawley rat liver microsomes in the presence of an NADPH-regenerating system. Six replicate incubations (final volume, 500 µL) were prepared in 100 mM potassium phosphate buffer (pH 7.4) containing microsomal protein (0.5 mg/mL), ArA (2 µM), and an NADPH-regenerating system composed of 1.3 mM NADP^+^, 3.3 mM glucose-6-phosphate, 3.3 mM MgCl_2_, and 0.4 U/mL glucose-6-phosphate dehydrogenase. A 200 µM ArA working solution was prepared in DMSO, giving a final organic solvent concentration of 1% (*v*/*v*) in the incubation mixture. The microsomes and NADPH-regenerating system were preincubated at 37 °C for 5 min in a shaking water bath with continuous agitation at 50 rpm, and the reaction was initiated by spiking the test compound into the mixtures. All subsequent incubations were maintained under the same conditions. At 0, 15, 30, 60, and 90 min, 50 µL aliquots were withdrawn and immediately quenched with 10 µL of the CBZ IS working solution and 190 µL of ice-cold acetonitrile. The samples were processed as described in Section 2.4 and analyzed by LC–MS/MS as described in Section 2.5.

The percentage of ArA remaining at each time point was calculated from the ArA concentration relative to that at 0 min. The apparent first-order depletion rate constant was obtained from the negative slope of the linear regression of the natural logarithm of the fraction remaining versus incubation time.

### 2.9. Data Analysis

Values were presented as the mean ± standard deviation (SD), except for T_max_, which was presented as the median with the observed range, and F, which was calculated from group mean values and was reported as a point estimate; the number of replicates was stated in the corresponding table or figure legend. Calibration curves and the apparent first-order rate constant for microsomal depletion were obtained by least-squares linear regression. Pharmacokinetic parameters were calculated by non-compartmental analysis as described in Section 2.7. No inferential statistical comparisons were performed. Figures were prepared using GraphPad Prism version 8.0.2 (GraphPad Software, San Diego, CA, USA).

## 3. Results and Discussion

### 3.1. Optimization of LC–MS/MS Conditions

MS conditions were optimized for the detection of ArA (Figure 1; C_45_H_72_O_12_; monoisotopic mass 804.50). Initially, signal intensities were compared between ESI in positive and negative modes. ArA exhibited greater signal intensity in the positive ionization mode, in which sodium adduct [M + Na]^+^ ions were predominantly detected at *m*/*z* 827.5. The predominance of the [M + Na]^+^ ion could be attributed to the absence of a strongly basic protonation site and the presence of numerous oxygen-donor groups in ArA, which could stabilize Na^+^ through multidentate coordination [8,17]. Accordingly, the [M + Na]^+^ ions were chosen as precursor ions and subsequently subjected to product ion scanning to identify suitable fragment ions for MRM analysis. As depicted in Figure 2, the most intense product ion was detected at *m*/*z* 765.5. Based on these results, the transitions from the precursor ions to their corresponding product ions (*m*/*z* 827.5 → 765.5) were selected for subsequent MRM mode optimization. Because a stable-isotope-labeled form of ArA was not commercially available, CBZ was selected as the IS on the basis of its reported stability in plasma [18], and its elution close to ArA under the optimized chromatographic conditions (Figure 3b,c). Its suitability for this assay was subsequently verified during validation (Section 3.2.4). CBZ also showed higher intensity in ESI positive mode compared to negative mode, with the protonated molecule [M + H]^+^ predominating at *m*/*z* 237.1. The product ion signal for CBZ was observed at *m*/*z* 194.1, which showed the fragmentation pattern consistent with that reported in a previous study [19] (Figure S1). To maximize signal intensity of ArA, the ionization source parameters were manually optimized with the gas flow held constant at 11 L/min. The conditions that produced the highest response were a nebulizer pressure of 60.0 psi, a gas temperature of 350 °C, and a capillary voltage of 4.00 kV (Table S1).

After establishing the optimal MS parameters, subsequent optimization of LC conditions was conducted. Among columns evaluated for LC optimization, Agilent Poroshell 120 EC-C18 column (2.7 µm, 4.6 × 50 mm) was selected for further investigation since it yielded superior peak resolution. The composition of the mobile phase, consisting of 10 mM ammonium formate in water and ACN, was optimized by adjusting the ACN proportion from 60% to 90%. According to the optimization results summarized in Table S2 and Figure S2, the peak area of ArA reached its maximum at an ACN content of 80%. Therefore, this composition was selected for subsequent analysis, considering key chromatographic parameters such as peak resolution, symmetry, signal intensity, and retention time.

### 3.2. Method Validation

#### 3.2.1. Selectivity and Carry-Over

The MRM chromatograms of ArA and IS are shown in Figure 3. In all six individual blank plasma lots, the responses within the retention windows of ArA and the IS did not exceed 20% of the mean ArA response at the LLOQ or 5% of the mean IS response, respectively, satisfiying the criteria specified in Section 2.6.1 (Figure 3a,b). ArA and IS exhibited retention times of 1.7 min and 1.5 min, respectively, which were consistently observed in plasma samples obtained after intravenous dosing of ArA (Figure 3b,c). Moreover, blank samples injected immediately after the ULOQ calibration standard showed responses below the same limits, indicating the absence of significant carry-over (Figure S3).

#### 3.2.2. Linearity and Lower Limit of Quantification

The calibration curves for ArA obtained with 1/*x* weighted least squares regression analyses were linear across the tested concentration range (2.0–2000 ng/mL), with the *r*^2^ higher than 0.996 (Figure S4). The LLOQ was established at 2.0 ng/mL with acceptable accuracy and precision, allowing reliable quantification of plasma concentrations over an adequate time course in the current pharmacokinetic study.

#### 3.2.3. Within- and Between-Run Accuracy and Precision

Following the confirmation of adequate selectivity and linearity for ArA quantification, the accuracy and precision of the analysis method were further evaluated based on RE and RSD values (Table 1). Within-run RE and RSD values for the LLOQ and QC samples ranged from −5.81% to 2.30% and 4.41% to 9.01%, respectively, meeting the acceptance criteria. Between-run accuracy and precision also remained within acceptable limits, with RE values from −3.20% to −0.10%, and RSD values between 7.20% and 10.38%. These findings confirm that the method exhibits acceptable accuracy and precision across multiple runs.

#### 3.2.4. Matrix Effect and Extraction Recovery

The matrix effect was evaluated using the accuracy and precision approach described in the ICH M10 guideline, in which low and high QC samples prepared in matrix from six individual sources are assessed against the accuracy and precision criteria (Table 2) [10]. The RE and RSD values ranged from −13.54% to 11.51% and from 0.40% to 11.85%, respectively, meeting the acceptance criteria in all six matrix sources at both QC levels. The extraction recovery ranged from 87.7 ± 5.1% to 96.5 ± 5.0% for ArA and was 82.3 ± 2.8% for the IS (Table S3), indicating consistent and reproducible extraction. Despite its structural dissimilarity to ArA, CBZ eluted close to ArA and showed reproducible extraction recovery. Together with the acceptable accuracy and precision obtained across six individual plasma sources and across analytical runs, these results support its fit-for-purpose use as a surrogate IS in the present assay.

#### 3.2.5. Pre- and Post-Preparative Stability

The stability of ArA was assessed under four experimental conditions (Table 3). Pre-preparative stability was evaluated under three conditions which included bench-top exposure at room temperature, long-term storage at −20 °C, and repeated freeze–thaw cycles. The RE values for each condition ranged from −3.35% to −1.66%, −2.37% to 0.34%, and −1.71% to −0.49%, respectively, with RSD values within acceptable limits. Additionally, post-preparative stability was assessed by storing the analytical samples in the autosampler at 10 °C for 24 h, yielding RE values between −5.22% and −1.75% and RSD values ranging from 5.02% to 7.16%. Collectively, these results demonstrate that ArA remained stable under all tested pre- and post-preparative conditions.

#### 3.2.6. Dilution Integrity

The dilution integrity was confirmed at 5-fold and 10-fold dilutions of 5000 ng/mL samples. The method demonstrated acceptable accuracy, with RE values of −4.56% and 2.33%, and precision, with RSD values of 6.00% and 4.57%, respectively (Table S4), supporting its suitability for quantifying samples above the calibration range.

### 3.3. Pharmacokinetic Profile of ArA

Following bioanalytical validation, the LC–MS/MS method was applied to characterize the pharmacokinetics of ArA after intravenous and oral administration in male Sprague–Dawley rats. The mean plasma concentration–time profiles and pharmacokinetic parameters are presented in Figure 4 and Table 4, respectively. After intravenous administration at 1 mg/kg, the terminal half-life and the plasma clearance were 0.78 ± 0.08 h and 26.06 ± 2.50 mL/min/kg, respectively. The steady-state volume of distribution was 921.75 ± 91.44 mL/kg, exceeding a reported rat total body water volume of 668 mL/kg [20], suggesting distribution into tissues rather than confinement to body water. The tissues responsible for this distribution were not identified, since tissue concentrations were not determined in the present study. For an anti-tuberculosis lead this is of particular interest, since efficacy is governed by drug concentrations at the site of infection rather than by plasma exposure alone, and ArA has been reported to retain activity against intracellular Mtb [9]. A tissue distribution study, with particular attention to the lung, would therefore be an informative next step, for which the present method could be adapted and cross-validated in tissue homogenates.

Following oral administration, ArA reached peak plasma concentrations rapidly, with a median T_max_ of 0.5 h (range, 0.5–1.0 h) at both 5 and 10 mg/kg. The mean C_max_ values were 438.62 ± 80.14 and 785.25 ± 18.77 ng/mL, and the corresponding AUC_inf_ values were 539.95 ± 225.61 and 915.28 ± 66.75 ng·h/mL, respectively. On a dose-normalized basis, both C_max_ and AUC_inf_ were of similar magnitude at the two dose levels. Individual values of AUC_inf_ and dose-normalized AUC_inf_ for all animals are provided in Table S5. The point estimates of absolute oral bioavailability were 16.8% and 14.2% at 5 and 10 mg/kg. ArA was therefore absorbed rapidly and produced consistent systemic exposure at both oral dose levels, with an absolute oral bioavailability of approximately 15% from the ethanol/PEG 400/normal saline vehicle. The terminal half-lives after oral administration, 0.69 ± 0.19 h at 5 mg/kg and 0.61 ± 0.09 h at 10 mg/kg, were similar to the value observed after intravenous administration.

The concentration–time profiles spanned the full validated range of the assay. Concentrations at the earliest sampling time after intravenous administration exceeded the ULOQ and were quantified after 5-fold dilution with blank rat plasma using the procedure validated in Section 3.2.6, whereas concentrations at the final sampling time were within an order of magnitude of the LLOQ of 2.0 ng/mL. The 1000-fold calibration range, together with the validated dilution procedure, therefore allowed both administration routes to be characterized using a single method. Sampling to 240 min corresponded to approximately five terminal half-lives, and AUC_last_ accounted for 98.0–98.6% of AUC_inf_ across the treatment groups, indicating that extrapolation beyond the last sampling time was minimal. The similarity of the terminal half-lives after intravenous and oral administration further indicates that the observed terminal phase reflects elimination rather than continued absorption, supporting the validity of the extrapolated AUC values used to estimate bioavailability. Nevertheless, several limitations of the present pharmacokinetic characterization should be noted. The study was exploratory in design and used three animals per group; the pharmacokinetic parameters and bioavailability estimates reported here are therefore associated with limited precision and should be regarded as preliminary. A single intravenous dose level and two oral dose levels were evaluated, which does not permit an assessment of dose proportionality.

Previous work in mice provided evidence of rapid oral absorption and measurable systemic exposure of ArA [9]. The present results extend those observations by providing quantitative estimates of absolute oral bioavailability and plasma pharmacokinetics in rats using a validated bioanalytical method. In vitro physicochemical and metabolic properties relevant to the observed oral exposure were evaluated in Section 3.4.

### 3.4. In Vitro Physicochemical and Metabolic Characterization

#### 3.4.1. Passive Membrane Permeability and Aqueous Solubility

The passive membrane permeability of ArA was evaluated using PAMPA, where those of nadolol, metoprolol, and ketoprofen were compared as reference compounds. The reference compounds exhibited the expected rank order of permeability (Table 5). ArA exhibited a *P*_app_ of 1.24 ± 0.20 × 10^−6^ cm/s, which was higher than that of metoprolol and similar to that of ketoprofen within the present assay. These findings suggest that ArA could partition into and permeate across a phospholipid membrane by passive diffusion despite its relatively high molecular weight and complex macrocyclic structure. PAMPA evaluates passive membrane permeability only and does not establish intestinal permeability or the extent of absorption in vivo, because it does not account for intestinal dissolution, transporter interactions, or presystemic metabolism. The present result should therefore be interpreted as indicating that passive membrane permeation is unlikely to be the principal barrier to the absorption of ArA, rather than as evidence of high intestinal permeability.

The apparent equilibrium solubility of ArA at 37 °C was 81.33 ± 8.02 µg/mL in PBS, 99.64 ± 1.24 µg/mL in DDW, and 105.06 ± 20.81 µg/mL in normal saline (Table S6). These values corresponded to approximately 0.10–0.13 mM, with no marked difference among the three media tested. Notably, these values were substantially lower than the ArA concentrations in formulations administered orally in pharmacokinetic studies; 1.25 and 2.5 mg/mL in a PEG400/normal saline/ethanol mixture for the 5 mg/kg and 10 mg/kg doses, respectively. This 12- to 25-fold difference between the equilibrium solubility and the administered concentration suggests that precipitation of ArA upon dilution in the aqueous gastrointestinal environment may contribute to the limited oral exposure of less than 20%. Precipitation was not, however, directly demonstrated under in vivo or simulated gastrointestinal conditions in the present study, and other factors that were not evaluated here—including intestinal metabolism, transporter-mediated efflux, and chemical instability in gastrointestinal fluids—cannot be excluded as contributors.

#### 3.4.2. Depletion in Rat Liver Microsomes

The apparent depletion of ArA was evaluated in pooled rat liver microsomes supplemented with an NADPH-regenerating system. ArA showed limited depletion over the 90 min incubation period, with 87.3 ± 4.5% remaining at the final time point (Figure S5). The apparent first-order depletion rate constant, calculated as the negative of the slope obtained by linear regression, was 0.00135 ± 0.00060 min^−1^. At a microsomal protein concentration of 0.5 mg/mL, this rate constant corresponded to an apparent in vitro intrinsic clearance (CL_int, in vitro_) of 2.70 ± 1.20 μL/min/mg protein. Applying scaling factors of 45 mg microsomal protein/g liver and 40 g liver/kg body weight yielded an apparent scaled intrinsic clearance (CL_int,scaled_) of 4.86 ± 2.16 mL/min/kg [20,21,22]. For a binding-uncorrected prediction, the ratio of the unbound fraction in blood to that in the microsomal incubation was set to unity in the well-stirred model, using a rat hepatic blood flow of 55.2 mL/min/kg [20]. This yielded a predicted hepatic blood clearance (CL_h,pred_) of approximately 4.5 mL/min/kg. Assuming a blood-to-plasma concentration ratio of unity, CL_h,pred_ corresponded to approximately 17% of the systemic plasma clearance (CL_p_) of 26.06 ± 2.50 mL/min/kg observed after intravenous administration (Table 4). Although this comparison is assumption-dependent and should be interpreted with caution, this difference suggests that NADPH-dependent microsomal metabolism alone may not fully account for the observed in vivo clearance of ArA. Other processes, including phase II or non-microsomal metabolism, extrahepatic metabolism, and renal or biliary excretion, may also contribute. Further investigation should first refine the comparison of CL_h,pred_ and CL_p_ by determining the unbound fractions in plasma and in the microsomal incubation, together with the blood-to-plasma concentration ratio. The routes of elimination should then be characterized by metabolite profiling in intact rat hepatocytes and by complementary uridine diphosphate glucuronic acid (UDPGA)-supplemented microsomal, S9, and cytosolic incubations. Finally, quantitative excretion and metabolite profiling in urine, bile, and feces following intravenous administration, including studies in bile duct-cannulated rats, would establish the relative contributions of metabolism and of renal and biliary excretion.

## 4. Conclusions

A sensitive and rapid LC–MS/MS method for the quantification of ArA in rat plasma was developed and validated with reference to the ICH M10 guideline. The method requires only 50 μL of plasma and a single protein-precipitation step, provides a 4 min isocratic run, covers a 1000-fold calibration range, and has validated dilution integrity. Its application to intravenous and oral pharmacokinetic studies provided a preliminary characterization of the plasma pharmacokinetics of ArA in rats, with an absolute oral bioavailability of approximately 15% at the two oral dose levels studied. Supporting in vitro assessments showed that ArA had high passive membrane permeability, limited aqueous solubility of approximately 0.1 mg/mL, and slow apparent depletion in rat liver microsomes. The limited solubility of ArA and its potential precipitation following oral administration may have contributed to the observed low oral bioavailability, although this possibility requires further investigation. Formulation strategies capable of improving and maintaining the dissolution and solubilization of ArA represent a logical next step in the preclinical development of ArA. Overall, the validated method provides a reliable analytical platform for further pharmacokinetic, formulation, and preclinical evaluation of ArA as a potential anti-tuberculosis agent.

## Figures and Tables

**Figure 1:**
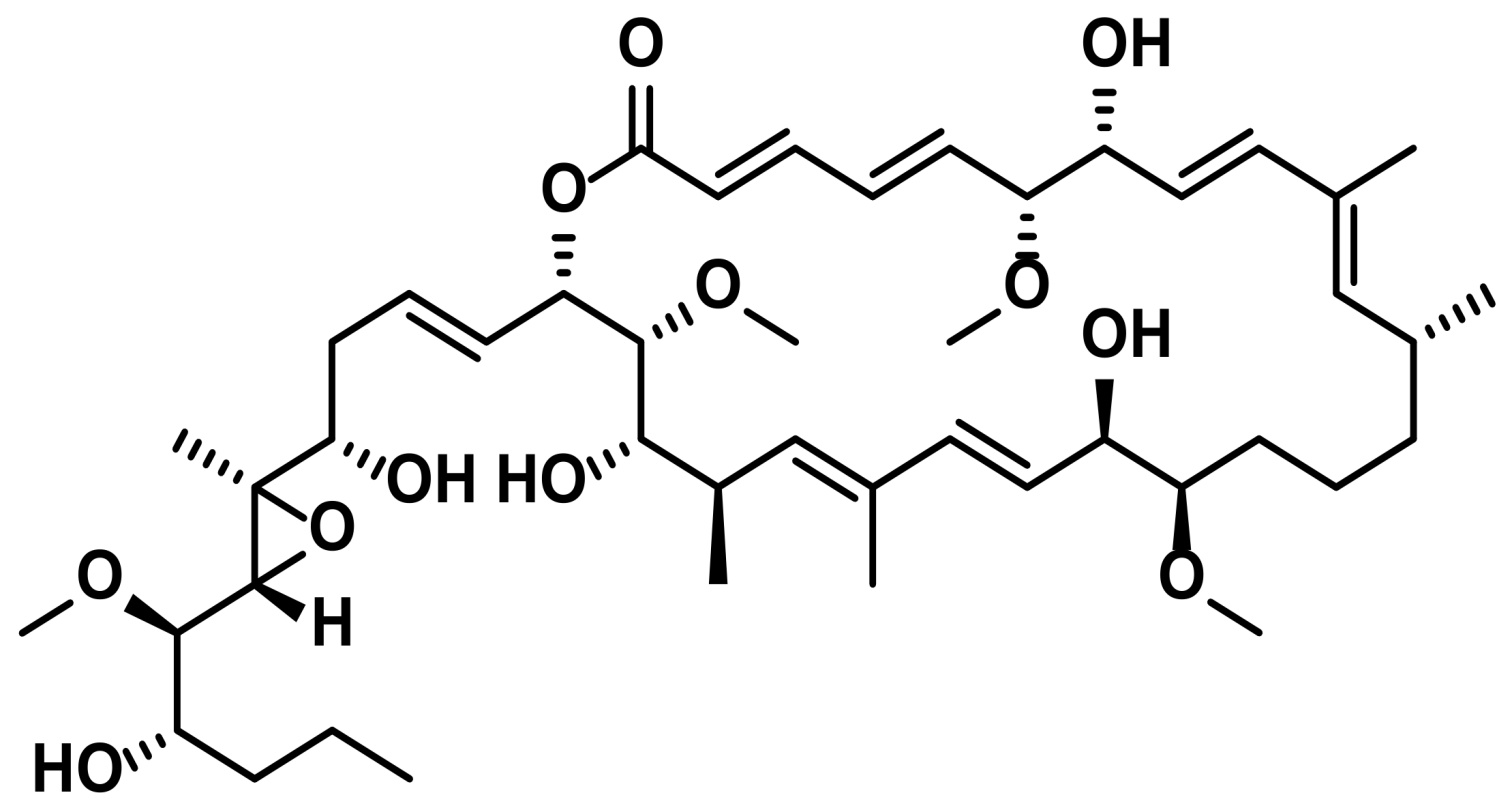
Chemical structure of arenicolide A (ArA).

**Figure 2:**
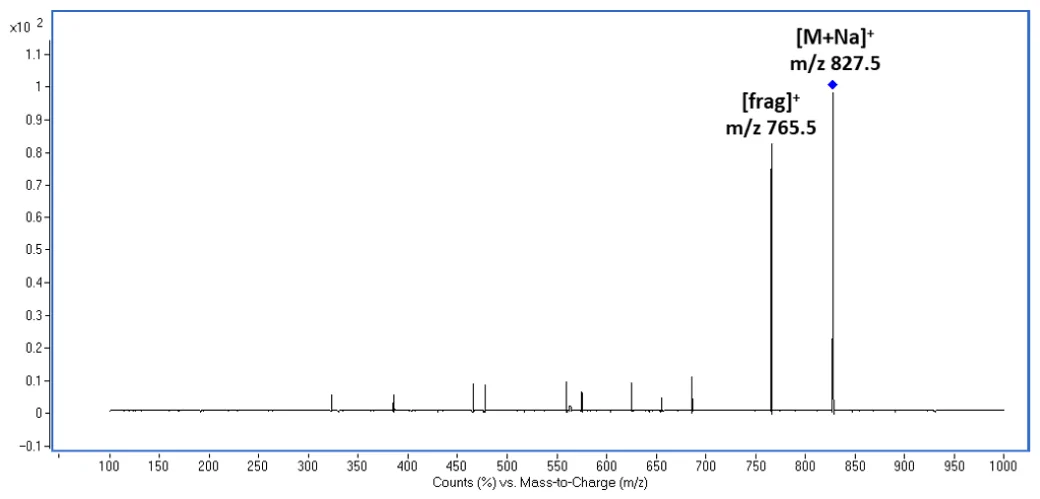
Product ion mass spectrum of ArA.

**Figure 3:**
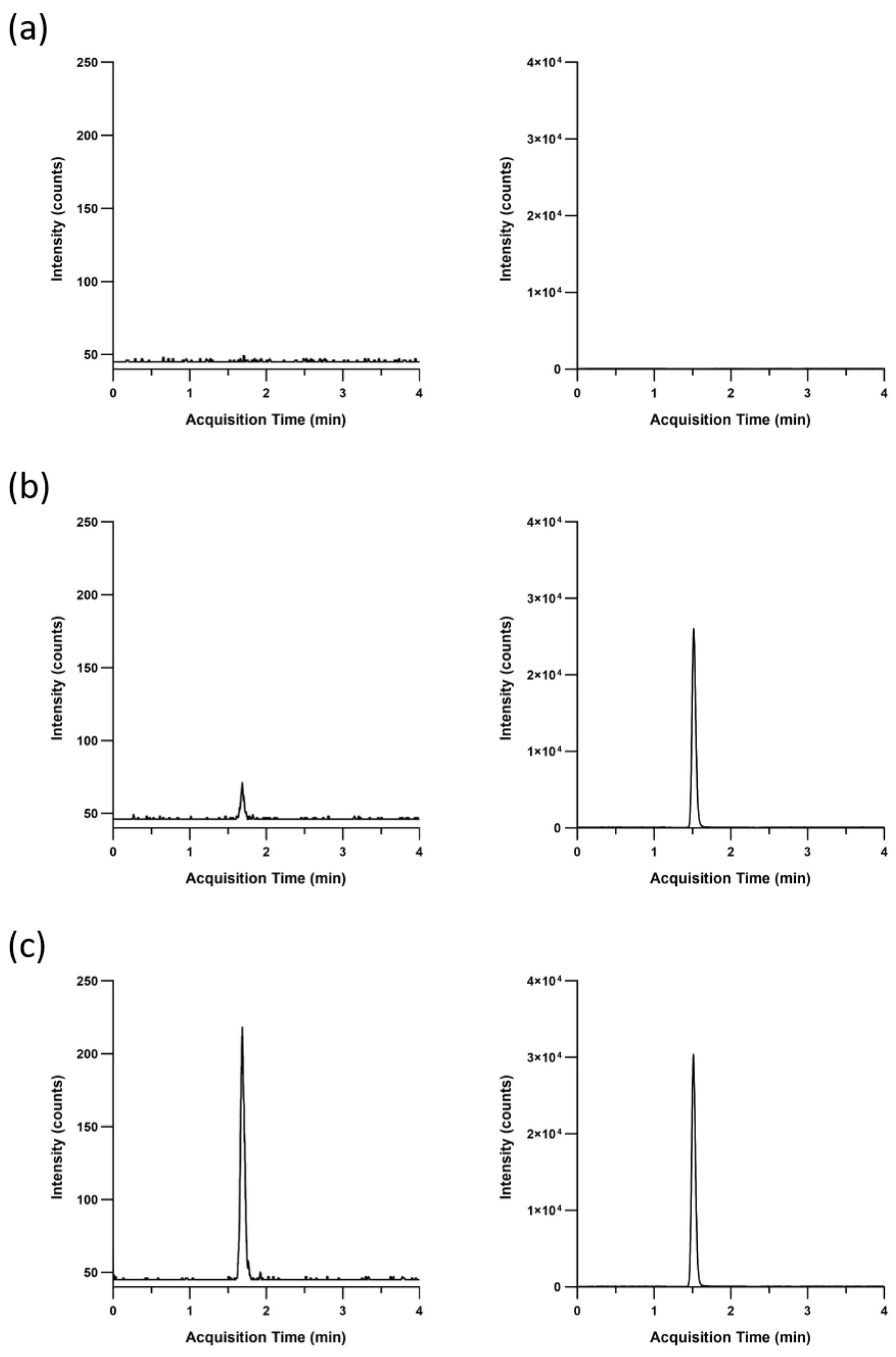
Representative chromatograms of ArA (left) and carbamazepine as internal standard (right) in rat plasma samples. (**a**) blank rat plasma sample, (**b**) the lower limit of quantification (2 ng/mL) sample of ArA and internal standard in rat plasma, (**c**) rat plasma sample collected at 240 min after an intravenous administration of ArA (dose: 1 mg/kg). Within each analyte channel, all panels are displayed on a common intensity scale.

**Figure 4:**
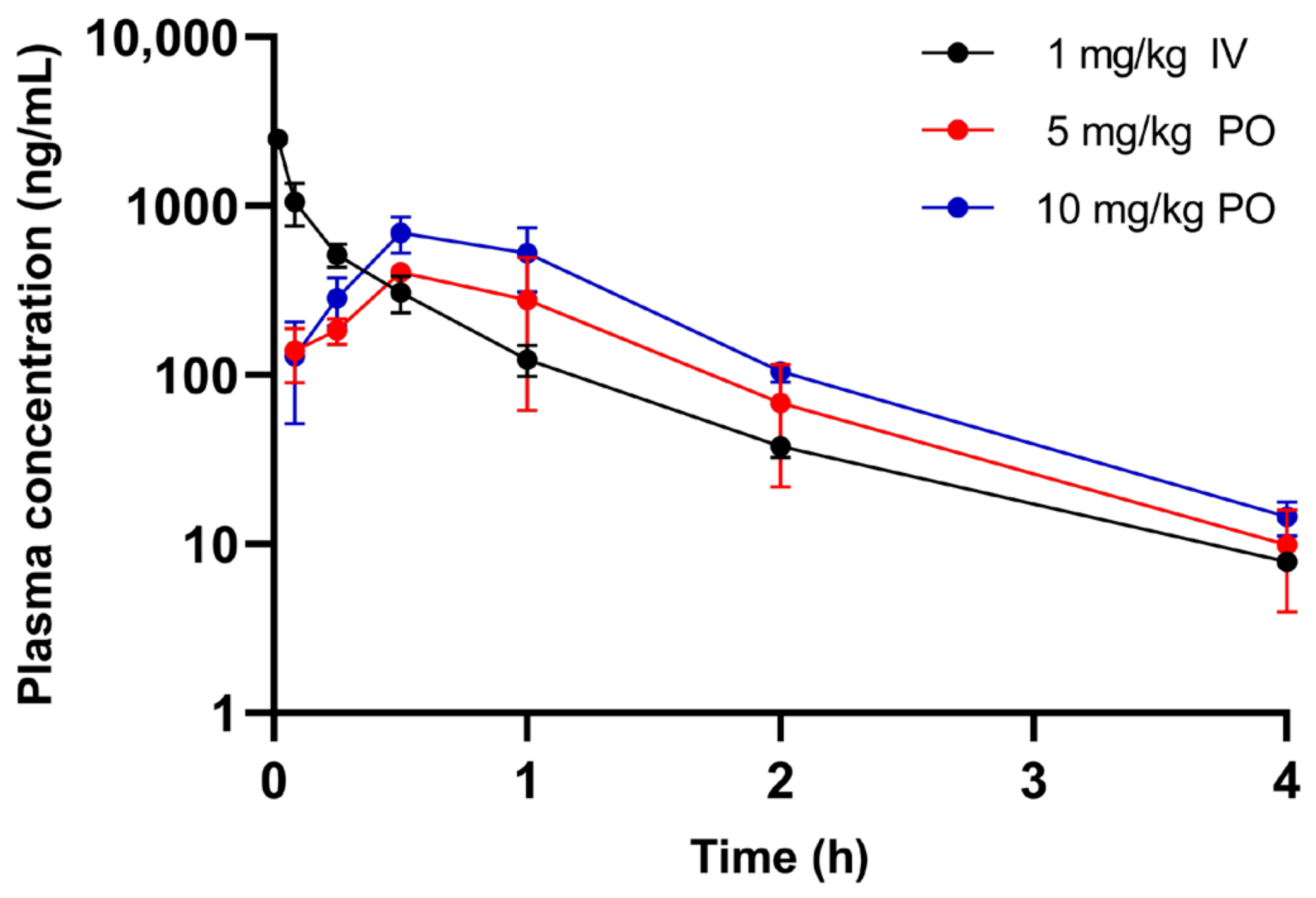
Mean plasma concentration–time profiles of ArA after intravenous administration (IV, 1 mg/kg) or oral administration (PO, 5 and 10 mg/kg) to male Sprague–Dawley rats. Data are presented as the mean ± standard deviation (SD) (*n* = 3).

**Table 1:** Within- and between-run accuracy and precision for ArA in rat plasma.

Nominal Concentration (ng/mL)	Within-Run (*n* = 6)			Between-Run (*n* = 18)		
	Concentration Determined (ng/mL)	RE ^a^ (%)	RSD ^b^ (%)	Concentration Determined (ng/mL)	RE (%)	RSD (%)
2.0	2.04	2.22	8.44	1.94	−3.20	10.38
5.0	5.12	2.30	7.03	4.99	−0.10	9.70
925	889.56	−3.83	9.01	909.44	−1.68	8.57
1850	1742.52	−5.81	4.41	1791.33	−3.17	7.20

^a^ RE (%) = ((determined concentration − nominal concentration)/nominal concentration) × 100. ^b^ RSD (%) = (standard deviation of determined concentrations/mean concentration) × 100.

**Table 2:** Matrix effect for ArA in rat plasma.

Nominal Concentration (ng/mL)	Matrix Effect (*n* = 3 Per Matrix Source, with 6 Different Sources)			
	Matrix Source	Concentration Determined (ng/mL)	RE (%)	RSD (%)
5.0 (Low QC)	1	4.67	−6.60	4.23
	2	5.31	6.16	0.40
	3	5.15	2.93	4.21
	4	4.93	−1.41	2.11
	5	4.41	−11.86	11.85
	6	4.84	−3.23	6.30
1850 (High QC)	1	1928.63	4.25	2.64
	2	1599.55	−13.54	7.20
	3	2046.01	10.59	3.90
	4	2062.98	11.51	2.98
	5	1909.55	3.22	3.72
	6	1730.62	−6.45	2.52

**Table 3:** Pre- and post-preparative stability of ArA (*n* = 4).

Nominal Concentration (ng/mL)	Bench-Top Stability ^a^			Long-Term Stability ^b^			Freeze–Thaw Stability ^c^			Autosampler Stability ^d^		
	Mean	RE (%)	RSD (%)	Mean	RE (%)	RSD (%)	Mean	RE (%)	RSD (%)	Mean	RE (%)	RSD (%)
5.0	4.91	−1.78	4.74	5.02	0.34	2.79	4.93	−1.38	7.31	4.74	−5.22	5.02
925	909.67	−1.66	0.38	909.59	−1.67	2.85	920.44	−0.49	1.52	908.78	−1.75	7.16
1850	1788.07	−3.35	2.26	1806.10	−2.37	4.28	1818.45	−1.71	1.15	1808.70	−2.23	5.54

^a^ After exposure at room temperature for 90 min. ^b^ After storage at −20 °C for 35 days. ^c^ After three freeze–thaw cycles. ^d^ After storage at 10 °C for 24 h in the autosampler.

**Table 4:** Pharmacokinetic parameters of ArA after intravenous or oral administration in Sprague–Dawley rats (*n* = 3).

Parameter	IV (1 mg/kg)	PO (5 mg/kg)	PO (10 mg/kg)
AUC_last_ (ng·h/mL)	634.97 ± 64.06	529.37 ± 228.22	902.31 ± 71.45
AUC_inf_ (ng·h/mL)	643.78 ± 64.61	539.95 ± 225.61	915.28 ± 66.75
t_1/2_ (h)	0.78 ± 0.08	0.69 ± 0.19	0.61 ± 0.09
CL_p_ (mL/min/kg)	26.06 ± 2.50	-	-
V_d,ss_ (mL/kg)	921.75 ± 91.44	-	-
C_max_ (ng/mL)	-	438.62 ± 80.14	785.25 ± 18.77
T_max_ (h) ^a^	-	0.5 (0.5–1.0)	0.5 (0.5–1.0)
F (%) ^b^	-	16.8	14.2

^a^ T_max_ is presented as the median (range). ^b^ Absolute bioavailability (F) was calculated as the ratio of the group mean dose-normalized AUC_inf_ values and is reported as a point estimate, since the intravenous and oral doses were administered to separate groups of animals.

**Table 5:** Apparent permeability (*P*_app_) values obtained in parallel artificial membrane permeability assay for ArA and the reference compounds.

Compound	Permeability Class ^a^	Human Fa ^b^	*P*_app_ (×10^−6^ cm/s) ^c^
Nadolol	Low	32–35%	0.004 ± 0.001
Metoprolol	Low/high boundary	90–95%	0.54 ± 0.05
Ketoprofen	High	≈100%	1.36 ± 0.10
ArA	-	-	1.24 ± 0.20

^a^ Permeability classification of the reference compounds was based on the literature [13,14,15,16]. ^b^ Reported human intestinal absorption [14,16]. ^c^ Values are presented as the mean ± SD of eight replicate wells (*n* = 8).

## Data Availability

The original contributions presented in this study are included in the article and Supplementary Materials. Further inquiries can be directed to the corresponding author.

## Supplementary Materials

The following supporting information can be downloaded at https://www.mdpi.com/article/10.3390/pharmaceutics18101205/s1, Figure S1: Proposed fragmentation patterns and product ion mass spectrum of carbamazepine (CBZ) as internal standard (IS). Figure S2: Representative chromatograms of ArA with various acetonitrile (ACN) contents in the mobile phase. Figure S3: Representative chromatograms of ArA (left) and IS (right) in blank rat plasma sample after the calibration standard at the upper limit of quantification. Figure S4: Representative calibration curve of ArA. Figure S5: Time course of ArA depletion in NADPH-supplemented rat liver microsomes.; Table S1: Optimization of ionization source settings. Table S2: Optimization of mobile phase composition. Table S3: Extraction recovery of ArA. Table S4: Dilution integrity of ArA. Table S5: Individual and mean area under the curve extrapolated to infinity (AUC_inf_) and dose-normalized AUC_inf_ of ArA in male Sprague–Dawley rats following intravenous (1 mg/kg) or oral (5 and 10 mg/kg) administration. Table S6: Apparent equilibrium solubility of ArA in phosphate-buffered saline (PBS, pH 7.4), double-distilled water (DDW), and normal saline determined by the saturation shake-flask method at 37 °C.

## Abbreviations

The following abbreviations are used in this manuscript:

ArA	Arenicolide A
ICH	International Council for Harmonisation
LC–MS/MS	Liquid chromatography–tandem mass spectrometry
MDR-TB	Multidrug-resistant tuberculosis
