# Supplementary material for: LC–MS/MS Quantification of the Anti-Tuberculosis Macrolide Arenicolide A in Rat Plasma: Method Validation and Pharmacokinetic Application
Source: Pharmaceutics. 2026 Sep 22;18(10):1205. doi: 10.3390/pharmaceutics18101205 (PMC13708135; doi:10.3390/pharmaceutics18101205)
Supplement: Supplementary file 1 [file pharmaceutics-18-01205-s001.zip › pharmaceutics-4552947-supplementary.pdf]

Supplementary Materials

LC–MS/MS Quantification of the Anti-Tuberculosis Macrolide Arenicolide A in Rat Plasma: Method Validation and Pharmacokinetic Application

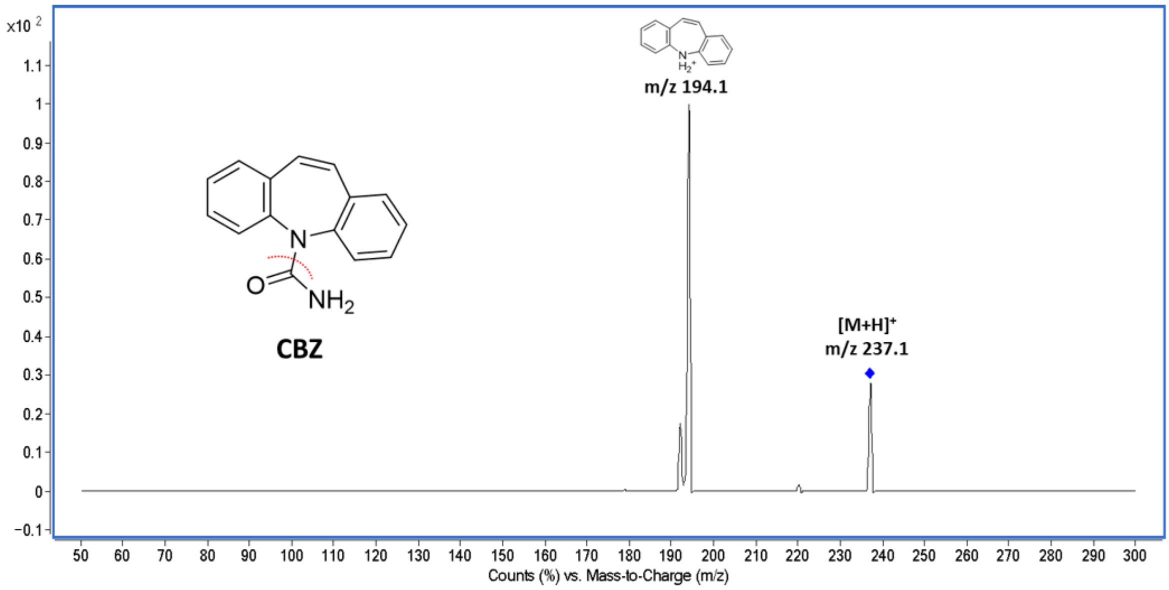

Figure S1. Proposed fragmentation patterns and product ion mass spectrum of carbamazepine (CBZ) as internal standard (IS).

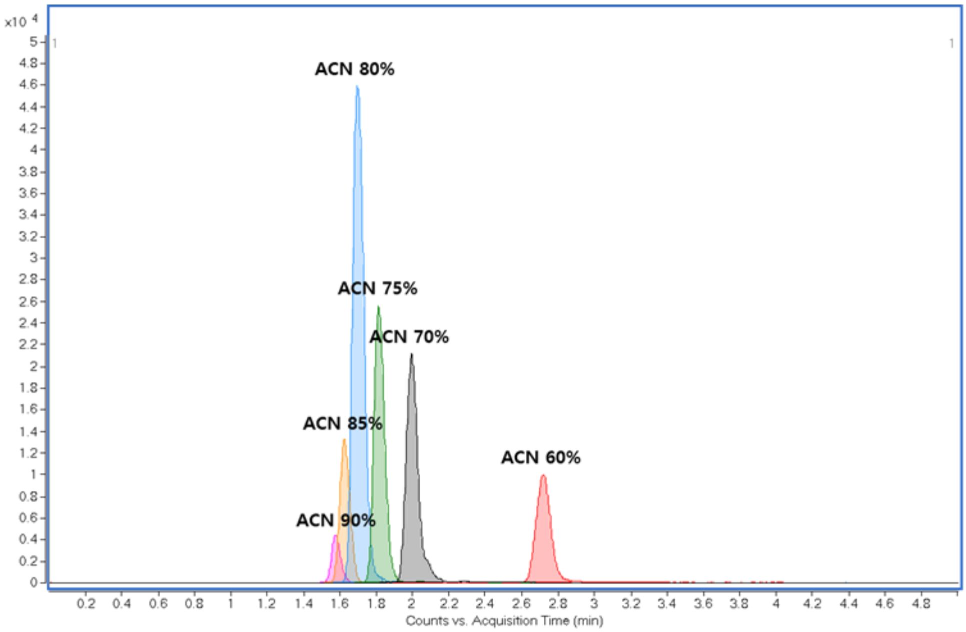

Figure S2. Representative chromatograms of arenicolide A (ArA) with various acetonitrile (ACN) contents in the mobile phase.

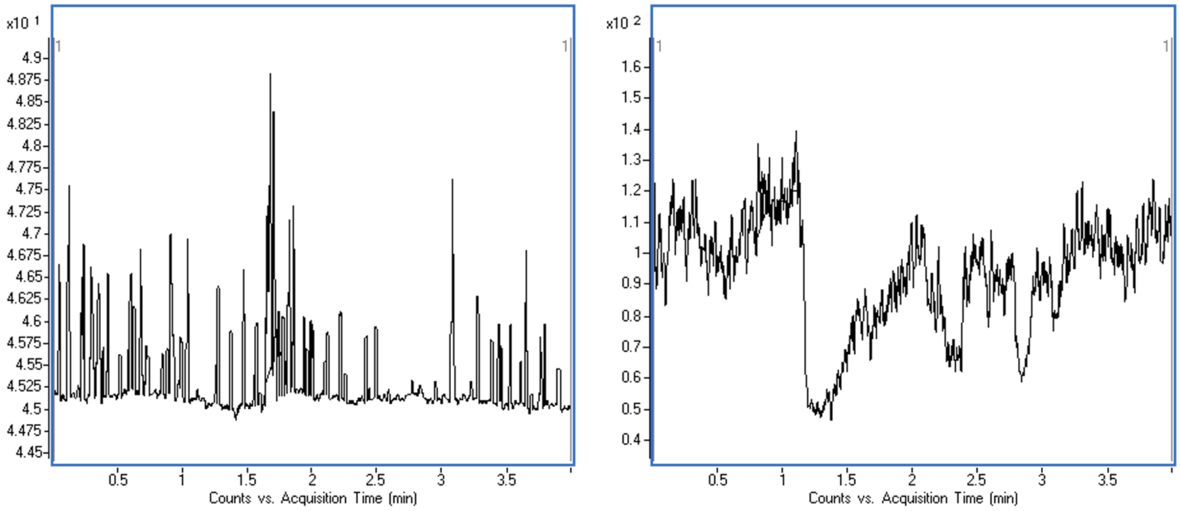

Figure S3. Representative chromatograms of ArA (left) and IS (right) in blank rat plasma sample after the calibration standard at the upper limit of quantification.

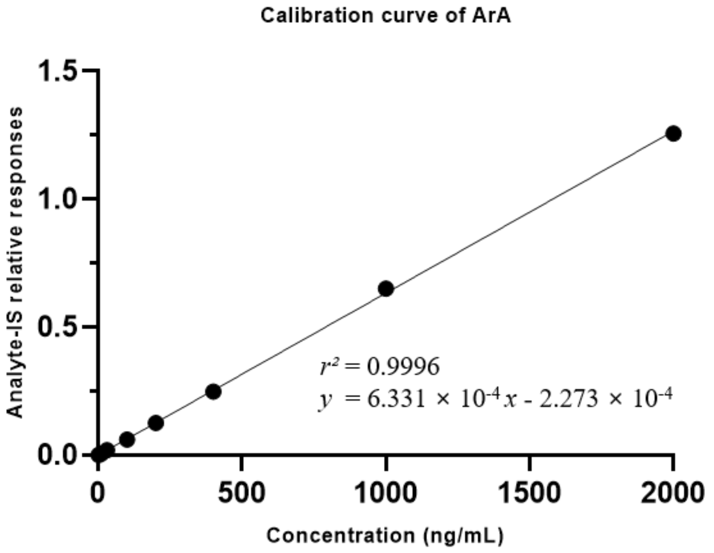

Figure S4. Representative calibration curve of ArA.

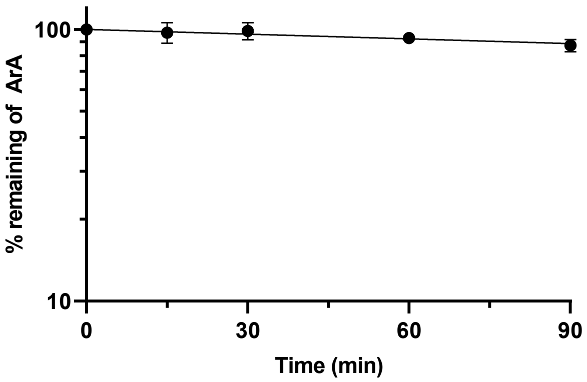

Figure S5. Time course of ArA depletion in NADPH-supplemented rat liver microsomes. The percentage of ArA remaining relative to the 0 min value is plotted on a semilogarithmic scale; each point represents the mean ± SD of six replicate incubations (n = 6). The solid line is the linear regression of the natural logarithm of the fraction remaining against incubation time, corresponding to a first-order depletion model.

Table S1. Optimization of ionization source settings.

Condition No.	Gas temperature (°C)	Nebulizer pressure (psi)	Capillary voltage (kV)	Fold-change in peak area[a]
1	300	30.0	4.00	1.00
2	300	15.0	4.00	0.74
3	300	50.0	4.00	1.07
4	325	50.0	4.00	1.18
5	350	50.0	4.00	1.31
6	350	30.0	4.00	1.20
7	350	30.0	3.00	1.05
8	350	50.0	3.00	1.21
9	350	50.0	5.00	1.31
10	350	60.0	4.00	1.38
11	350	60.0	5.00	1.35
12	350	60.0	6.00	1.34

[a] Compared to condition No. 1.

Table S2. Optimization of mobile phase composition.

Condition No.	% of ACN	Fold-change in peak area[a]
1	60	1.00
2	70	1.72
3	75	1.89
4	80	3.58
5	85	0.92
6	90	0.28

[a] Compared to condition No. 1.

Table S3. Extraction recovery of ArA. Data are presented as the mean ± standard deviation (SD) (n = 3).

Nominal Concentration (ng/mL)	Extraction Recovery (%)[a]
**ArA**	
5.0	87.7 ± 5.1
925	89.4 ± 2.9
1850	96.5 ± 5.0
**IS (CBZ in processed sample)**	
8.0	82.3 ± 2.8

[a] Extraction recovery (%) = ((peak area of analyte spiked before extraction)/(peak area of analyte spiked after extraction)) × 100.

Table S4. Dilution integrity of ArA (n = 6).

Nominal concentration (ng/mL)	Concentration Determined (ng/mL)	RE (%)	RSD (%)
5000<br>(Dilution factor: 5)	4772.02	-4.56	6.00
5000<br>(Dilution factor: 10)	5116.70	2.33	4.57

Table S5. Individual and mean area under the curve extrapolated to infinity (AUC_{inf}) and dose-normalized AUC_{inf} of ArA in male Sprague–Dawley rats following intravenous (1 mg/kg) or oral (5 and 10 mg/kg) administration (n = 3 per group).

Group	Individual AUC_{inf} (ng·h/mL)	Individual dose-normalized AUC_{inf} (ng·h/mL)/(mg/kg)	Mean dose-normalized AUC_{inf} ± SD (RSD, %)
IV, 1 mg/kg	595.04; 619.24; 717.06	595.04; 619.24; 717.06	643.78 ± 64.61 (10.0)
PO, 5 mg/kg	464.42; 793.63; 361.80	92.88; 158.73; 72.36	107.99 ± 45.12 (41.8)
PO, 10 mg/kg	856.10; 987.64; 902.10	85.61; 98.76; 90.21	91.53 ± 6.68 (7.3)

Table S6. Apparent equilibrium solubility of ArA in phosphate-buffered saline (PBS, pH 7.4), double-distilled water (DDW), and normal saline determined by the saturation shake-flask method at 37 °C. Data are presented as the mean ± SD (n = 3).

Solvent	Solubility (μg/mL)
PBS	81.33 ± 8.02
DDW	99.64 ± 1.24
Normal saline	105.06 ± 20.81
